# Healthcare Resource Utilization and Costs Among Patients With Gastroesophageal Reflux Disease, Barrett’s Esophagus, and Barrett’s Esophagus-Related Neoplasia in the United States

**DOI:** 10.36469/001c.68191

**Published:** 2023-03-03

**Authors:** Prateek Sharma, Gary W. Falk, Menaka Bhor, A. Burak Ozbay, Dominick Latremouille-Viau, Annie Guerin, Sherry Shi, Margaret M. Elvekrog, Paul Limburg

**Affiliations:** 1 University of Kansas School of Medicine and VA Medical Center, Kansas City, Missouri; 2 University of Pennsylvania Perelman School of Medicine, Philadelphia, Pennsylvania; 3 Exact Sciences, Madison, Wisconsin; 4 Analysis Group, Montréal, Québec, Canada

**Keywords:** Barrett’s esophagus, gastroesophageal reflux disease, esophageal adenocarcinoma, healthcare resource utilization, direct healthcare costs

## Abstract

**Background:** Gastroesophageal reflux disease (GERD) is a risk factor for Barrett’s esophagus (BE) and BE-related neoplasia (BERN). **Objectives:** This study aimed to evaluate healthcare resource utilization (HRU) and costs associated with GERD, BE, and BERN in the United States. **Methods:** Adult patients with GERD, nondysplastic BE (NDBE), and BERN (including indefinite for dysplasia [IND], low-grade dysplasia [LGD], high-grade dysplasia [HGD] or esophageal adenocarcinoma [EAC]), were identified from a large US administrative claims database, the IBM Truven Health MarketScan® databases (Q1/2015-Q4/2019). Patients were categorized into the corresponding mutually exclusive EAC-risk/diagnosis cohorts based on the most advanced stage from GERD to EAC using diagnosis codes in medical claims. Disease-related HRU and costs (2020 USD) were calculated for each cohort. **Results:** Patients were categorized into the following EAC-risk/diagnosis cohorts: 3 310 385 into GERD, 172 481 into NDBE, 11 516 into IND, 4332 into LGD, 1549 into HGD, and 11 676 into EAC. Disease-related annual mean number of inpatient admissions, office visits, and emergency department visits by cohort were 0.09, 1.45, and 0.19 for GERD; 0.08, 1.55, and 0.10 for NDBE; 0.10, 1.92, and 0.13 for IND; 0.09, 2.05, and 0.10 for LGD; 0.12, 2.16, and 0.14 for HGD; and 1.43, 6.27, and 0.87 for EAC. Disease-related annual mean total healthcare costs by cohort were 6955forGERD,8755 for NDBE, 9675forIND,12 241 for LGD, 24 239forHGD,and146 319 for EAC. **Discussion:** Patients with GERD, BE, and BERN had important HRU and costs, including inpatient admissions and office visits. As patients progressed to more advanced stages, there was substantially higher disease-related resource utilization, with associated costs being 16 times higher in patients with EAC than those with NDBE. **Conclusions:** Findings suggest the need for early identification of high-risk individuals prior to progression to EAC to potentially improve clinical and economic outcomes in this population.

## BACKGROUND

Gastroesophageal reflux disease (GERD), characterized by heartburn and regurgitation, is one of the most common gastrointestinal disorders,^1^ and is a risk factor for Barrett’s esophagus (BE), a known risk factor for esophageal adenocarcinoma (EAC). Barrett’s esophagus is defined by the presence of columnar epithelium of the lower esophagus with a metaplastic change in the lining of the esophagus characterized with goblet cells.^2^ Approximately 25% to 35% of adults in the United States have GERD and, of these patients, 10% to 20% develop BE with a prevalence of 1.6% in the general population.^3^ Barrett’s esophagus is the earliest phase of the carcinogenesis pathway,^4^ and the metaplastic changes that result in BE can progress to dysplasia (4.3% incidence rate for low-grade dysplasia [LGD]; 0.9% incidence rate for high-grade dysplasia [HGD]) and ultimately esophageal adenocarcinoma (EAC; 0.13%-0.3% incidence rate).^3,5,6^ Barrett’s esophagus remains the only known precursor to EAC,^7,8^ one of the most lethal cancers in the United States with a median survival time of 15 months.^9^

In addition to the important clinical burden associated with EAC and its antecedent phases, the associated economic burden can be substantial.^10–12^ In 2018, the annual healthcare expenditures for esophageal disorders exceeded $12 billion in the United States.^10^ One study estimated the direct medical cost of a hypothetical population of 25 000 patients with GERD to be $8.6 million higher than for those without GERD.^11^

As the incidence of BE and EAC continues to increase over time,^8,13–15^ the associated economic burden is likely to increase, necessitating an up-to-date evaluation. In addition, progress in the clinical management of BE over the past 2 decades warrants an updated comprehensive assessment of this population’s economic burden to capture the variability in costs of care by disease stage. To that end, the aim of this study was to evaluate healthcare resource utilization (HRU) and direct healthcare costs associated with GERD, BE, and BERN. This would provide an understanding of the contemporary economic outcomes in the United States among these patients by stage of disease to inform economic models, which may serve as a benchmark to assist in improving the clinical management of BE.

## METHODS

### Data Source

Patients were identified from a large US administrative claims database, the IBM Truven Health MarketScan® Commercial Claims and Encounters, and the Medicare Supplemental and Coordination of Benefits databases (January 1, 2015–December 31, 2019). The Commercial Claims and Encounters database is a collection of paid claims generated by nearly 51 million employees and their dependents per year with employer-sponsored insurance and enrolled in a variety of over 150 different health plans including fee-for-service and managed care plans. The Medicare Supplemental and Coordination of Benefits database consists of claims for Medicare-eligible retirees and their dependents with employer-sponsored supplemental insurance. The databases include detailed information on demographics, health plan, medical care received across different settings, such as inpatient (IP) and outpatient (OP) services, diagnoses, procedures, and prescription medications. All census regions are well represented, although there is a slightly higher representation from the South and North Central (Midwest) regions. All data are de-identified and comply with the Health Insurance Portability and Accountability Act (HIPAA); therefore, no institutional review board approval was required.

### Study Design and Sample Selection

This study used a retrospective cohort design whereby adult patients were classified into 6 mutually exclusive EAC-risk/diagnosis cohorts based on their most advanced stage of disease from GERD to BERN (ie, IND, LGD, HGD, EAC)^16–18^:

1. GERD cohort: patients had ≥2 medical claims with a diagnosis for GERD on distinct calendar dates, or had ≥1 medical claim with a diagnosis for GERD and ≥1 treatment for GERD preceded by a GERD diagnosis
2. Nondysplastic BE (NDBE) cohort: patients had ≥1 medical claim with a diagnosis for NDBE
3. IND (indefinite for dysplasia) cohort: patients had ≥1 medical claim with a diagnosis for BE with IND
4. LGD cohort: patients had ≥1 medical claim with a diagnosis for BE with LGD
5. HGD cohort: patients had ≥1 medical claim with a diagnosis for BE with HGD
6. EAC cohort: patients had ≥1 medical claim with a diagnosis for EAC

**Figure 1.**
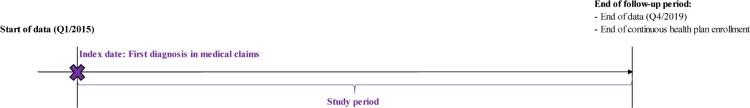
Study Design

The first confirmed diagnosis of GERD, NDBE, IND, LGD, HGD, or EAC marked the index date for patients in each of the 6 cohorts. Patients were required to have at least 1 month of continuous health plan enrollment (medical and pharmacy) on or after the index date. The study period spanned from the index date to the end of continuous health plan enrollment or data availability, whichever occurred first (Figure 1).

### Study Measures and Outcomes

Patient demographic and clinical characteristics, and all-cause and disease-related HRU and direct healthcare costs, were measured by EAC risk/diagnosis cohort from GERD to EAC. Disease-related outcomes were identified using claims with a diagnosis code, procedure code for a diagnostic test or treatment, or National Drug Code for a treatment for GERD, NDBE, IND, LGD, HGD, or EAC. Healthcare resource utilization included IP admissions, emergency department (ED) visits, and days with OP services (including laboratory tests, imaging, mental health services, drug administration, skilled nursing facilities, and home care/hospice services in addition to office visits [day with office visits excluded other type of OP services during that day]). Direct healthcare costs included total (medical and pharmacy), medical (IP, ED, OP), and pharmacy costs, as well as costs associated with disease-related esophagogastroduodenoscopy (EGD). Costs were evaluated from a payer perspective (ie, amounts reimbursed by the health plan and coordination of benefit), adjusted for inflation using the US Medical Care component of the Consumer Price Index, and reported in 2020 US dollars.^19^

### Statistical Analyses

This study was descriptive in nature and reported unadjusted estimates by cohort. The proportion of patients with at least 1 all-cause and disease-related HRU event and the annual mean number of all-cause and disease-related HRU events per patient, as well as the all-cause and disease-related annual mean healthcare costs per patient and mean disease-related EGD costs at the EGD event level were described by cohort using means, SD, 95% confidence intervals (CIs), and medians for continuous variables, and frequency counts and proportions for categorical variables. All analyses were performed using SAS® Enterprise Guide 7.1 (SAS Institute, Inc., Cary, North Carolina).

## RESULTS

### Study Sample

**Figure 2.**
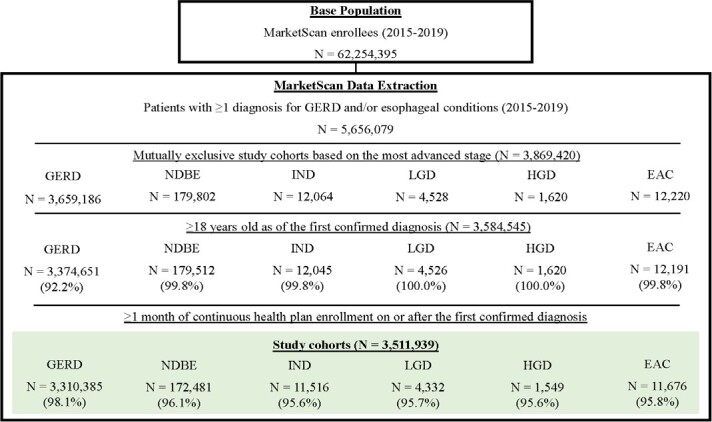
Sample Selection by Cohort from GERD to EAC Disease Stage Abbreviations: EAC, esophageal adenocarcinoma; GERD, gastroesophageal reflux disease; HGD, high-grade dysplasia; IND, indefinite for dysplasia; LGD, low-grade dysplasia; NDBE, nondysplastic Barrett’s esophagus.

Of 5 656 079 patients with at least 1 diagnosis for GERD and/or esophageal conditions in the database (2015-2019), the study sample comprised 3 511 939 patients who met the eligibility criteria: 3 310 385 in the GERD cohort, 172 481 in the NDBE cohort, 11 516 in the IND cohort, 4332 in the LGD cohort, 1549 in the HGD cohort, and 11 676 in the EAC cohort based on their most advanced stage of disease (Figure 2).

### Patient Characteristics

The cohorts included a clinically representative population of patients with EAC and its precursors. The mean (SD) age at index date was 51.0 (15.0) years for GERD patients, 57.1 (12.1) years for NDBE, and ranged between 58.1 (11.7) and 62.8 (12.0) years for the BERN cohorts. The majority of patients were female in the GERD cohort (59.6%) and male in the NDBE and BERN cohorts (56.1%-76.8%), and resided in the South (35.1%-49.0%) and Midwest/North Central (21.1%-27.8%) US regions. Patients had a mean (SD) of 16.6 (14.0) to 22.9 (15.9) months of follow-up during the study period across cohorts, and 49.3% to 67.0% of patients had at least 12 months of follow-up from the index date (Table 1).

**Table 1.** Patient Characteristics by Cohort from GERD to EAC Disease Stage

	**GERD** **(n = 3 310 385)**	**NDBE** **(n = 172 481)**	**IND (n = 11 516)**	**LGD (n = 4332)**	**HGD (n = 1549)**	**EAC (n = 11 676)**
Age (y); mean ± SD [median]	51.02 ± 14.97 [52.00]	57.05 ± 12.16 [58.00]	58.07 ± 11.74 [58.00]	60.12 ± 11.27 [60.00]	61.29 ± 10.93 [61.00]	62.79 ± 12.03 [62.00]
Male, n (%)	1 337 970 (40.4)	96 769 (56.1)	6770 (58.8)	2898 (66.9)	1190 (76.8)	8740 (74.9)
Region of residence, n (%)						
South	1 621 109 (49.0)	71 407 (41.4)	4047 (35.1)	1659 (38.3)	571 (36.9)	4469 (38.3)
Midwest/North Central	699 450 (21.1)	38 393 (22.3)	3200 (27.8)	1072 (24.7)	380 (24.5)	3135 (26.8)
Northeast	578 927 (17.5)	41 045 (23.8)	2775 (24.1)	1078 (24.9)	413 (26.7)	2624 (22.5)
West	405 824 (12.3)	21 452 (12.4)	1479 (12.8)	519 (12.0)	185 (11.9)	1434 (12.3)
Unknown	5075 (0.2)	184 (0.1)	15 (0.1)	4 (0.1)	0 (0.0)	14 (0.1)
Duration of study period (mo), mean ± SD [median]	22.92 ± 15.94 [19.74]	18.86 ± 13.33 [15.63]	17.64 ± 12.83 [14.57]	18.19 ± 12.92 [15.00]	18.05 ± 13.21 [14.24]	16.59 ± 13.92 [11.91]
≥12 mo, n (%)	2 218 827 (67.0)	104 101 (60.4)	6571 (57.1)	2589 (59.8)	885 (57.1)	5756 (49.3)
Charlson Comorbidity Index,^a^ mean ± SD	0.99 ± 1.66	1.06 ± 1.71	1.08 ± 1.73	1.14 ± 1.80	1.29 ± 1.88	5.63 ± 2.73

Abbreviations: EAC, esophageal adenocarcinoma; GERD, gastroesophageal reflux disease; HGD, high-grade dysplasia; IND, indefinite for dysplasia; LGD, low-grade dysplasia; NDBE, nondysplastic Barrett’s esophagus.

^a^Measured during the study period, including the index date. Quan H, Sundararajan V, Halfon P, et al. Coding algorithms for defining comorbidities in ICD-9-CM and ICD-10 administrative data. *Medical Care*. 2005;43:1130-1139; and Quan H, Li B, Couris CM, et al. Updating and validating the Charlson comorbidity index and score for risk adjustment in hospital discharge abstracts using data from 6 countries. *Am J Epidemiol.* 2011;173(6):676-682.

### Healthcare Resource Utilization

**Figure 3.**
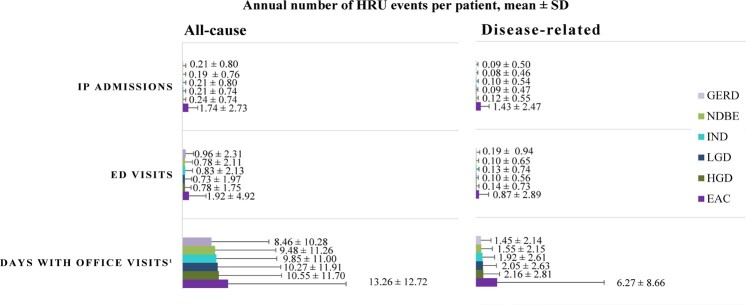
Annual Mean Number of HRU Events by Cohort from GERD to EAC Disease Stage Abbreviations: EAC, esophageal adenocarcinoma; ED, emergency department; GERD, gastroesophageal reflux disease; HGD, high-grade dysplasia; HRU, healthcare resource utilization; IND, indefinite for dysplasia; IP, inpatient; LGD, low-grade dysplasia; NDBE, nondysplastic Barrett’s esophagus. Office visits excluded days with outpatient services such as laboratory tests, imaging, mental health services, drug administration, skilled nursing facilities, and home care services. See **Supplementary Table S1** for the full list of outpatient services.

During the study period, the proportion of patients requiring all-cause IP admissions and ED visits increased with each progressive disease stage from NDBE to EAC, and nearly all patients, regardless of the cohort, had at least 1 all-cause office visit (Table 2). Disease-related HRU followed a similar trend, with 53.3% of patients in the EAC cohort having at least 1 disease-related IP admission during the study period, relative to only 7.3% of patients in the NDBE cohort (Table 2). Notably, disease-related IP utilization was observed to be numerically 18 times higher among patients with EAC than those with NDBE, with an annual mean (SD) number of 1.4 (2.5) disease-related IP admissions for patients with EAC, which represented 82.2% of all-cause IP admissions (Figure 3). Similarly, the proportion of patients with at least 1 disease-related ED visit during the study period ranged from 8.0% in the NDBE cohort to 29.5% in the EAC cohort (Table 2). The majority of patients, regardless of the cohort, had at least 1 day with disease-related office visits during the study period, with an annual mean (SD) ranging from 1.6 (2.2) days with disease-related office visits for those with NDBE to 6.3 (8.7) days for patients with EAC **(Figure 3; Supplementary Table S1**).

**Table 2.** Proportion of Patients With ≥1 HRU Event During the Study Period from GERD to EAC Disease Stage

	**GERD** **(n = 3 310 385)**	**NDBE** **(n = 172 481)**	**IND** **(n = 11 516)**	**LGD** **(n = 4332)**	**HGD** **(n = 1549)**	**EAC** **(n = 11 676)**
Duration of study period (mo), mean ± SD [median]	22.92 ± 15.94 [19.74]	18.86 ± 13.33 [15.63]	17.64 ± 12.83 [14.57]	18.19 ± 12.92 [15.00]	18.05 ± 13.21 [14.24]	16.59 ± 13.92 [11.91]
All-cause						
IP admissions	18.5%	15.4%	15.5%	16.4%	20.7%	61.8%
ED visits	49.4%	41.0%	40.2%	38.6%	39.7%	56.1%
Days with OP services^a^	99.7%	99.9%	99.9%	100.0%	99.8%	99.3%
Days with office visits^b^	95.1%	94.2%	93.5%	94.7%	94.6%	95.2%
Disease-related						
IP admissions	9.1%	7.3%	8.4%	8.3%	10.8%	53.3%
ED visits	13.4%	8.0%	8.9%	8.2%	9.7%	29.5%
Days with OP services^a^	95.9%	98.7%	98.8%	99.4%	99.2%	97.0%
Days with office visits^b^	77.7%	71.5%	76.7%	78.3%	75.9%	82.1%

Abbreviations: EAC, esophageal adenocarcinoma; ED, emergency department; GERD, gastroesophageal reflux disease; HGD, high-grade dysplasia; HRU, healthcare resource utilization; IND, indefinite for dysplasia; IP, inpatient; LGD, low-grade dysplasia; NDBE, nondysplastic Barrett’s esophagus; OP, outpatient; SNF, skilled nursing facilities.

^a^OP services included office visits, laboratory tests, imaging, mental health services, drug administration, SNF, or home care services not received in an IP or ED setting.

^b^Office visits excluded days with OP services such as laboratory tests, imaging, mental health services, drug administration, SNF, and home care services.

### Direct Healthcare Costs

**Figure 4.**
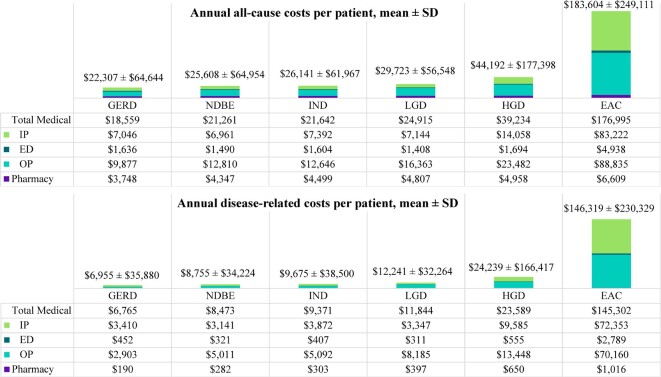
Annual Direct Healthcare Costs by Cohort from GERD to EAC Disease Stage (USD 2020) Abbreviations: EAC, esophageal adenocarcinoma; ED, emergency department; GERD, gastroesophageal reflux disease; HGD, high-grade dysplasia; HRU, healthcare resource utilization; IND, indefinite for dysplasia; IP, inpatient; LGD, low-grade dysplasia; NDBE, nondysplastic Barrett’s esophagus, OP, outpatient.

Consistent with the trends observed in HRU, healthcare costs increased with disease severity whereby patients with NDBE incurred a mean (SD; 95% CI) of $8755 ($34 224; $8593-$8917) in disease-related total costs per patient per year (PPPY; 34.2% of all-cause total costs) while those with EAC incurred $146 319 ($230 329; 142 141-$150 497) PPPY (79.7% of all-cause total costs). Medical costs were the major driver of the total direct healthcare costs, representing a proportion of more than 95% of disease-related total costs across all cohorts. Disease-related medical costs ranged from a mean (SD; 95% CI) of $8473 ($34 165; $8312-$8634) PPPY among patients with NDBE to $145 302 ($230 112; $141 128-$149 476) PPPY among those with EAC. Of those medical costs, IP and OP were the main cost drivers. Patients with NDBE and EAC incurred a mean (SD; 95% CI) of $3141 ($30 959; $2995-$3287) and $72 353 ($185 685; $68 985-$75 721) in disease-related IP costs PPPY and $5011 ($11 780; $4955-$5067) and $70 160 ($109 069; $68 182-$72 138) in disease-related OP costs PPPY, respectively. Pharmacy costs were relatively negligible though they followed a similar increasing trend with increasing disease severity (**Figure 4; Supplementary Table S2**). The mean (SD) costs associated with disease-related EGD at the event level were $2793 ($2520) per EGD.

## DISCUSSION

In this study, we found that patients across all EAC risk/diagnosis cohorts had substantial disease-related HRU with an annual mean of 1 to 2 office visits for patients with GERD, NDBE, or dysplasia, which reached 6 for those with EAC. Furthermore, more than half of the patients with EAC required disease-related hospitalization at least once during the study period. As patients progressed from GERD to EAC, they also incurred substantially higher disease-related healthcare costs, with a numeric annual mean difference of nearly $140 000 per patient between those with EAC and those with NDBE. Total medical costs were driven primarily by IP and OP services, with a steep increase in the contribution of IP costs in advanced disease stages. It is worth noting that a high variability was observed relative to the mean healthcare costs across patients by cohort given that the occurrence of HRU events varied by patient.

The burden associated with esophageal disorders has been well characterized in the United States, with healthcare expenditures exceeding $12 billion in 2018.^10^ However, a comprehensive assessment of the real-world HRU and costs associated with EAC, one of the most lethal cancers in the United States,^9^ and its precursor stages has not been recently and comprehensively evaluated. Even the earliest stage of disease, GERD, can be associated with substantial costs,^11,20^ and increasing severity of GERD symptoms results in higher HRU, including IP admissions, ED visits, and physician office visits.^21^ The current study sheds light on the wide range of healthcare services used by patients across all cohorts, including outpatient visits. Results show a gradual numeric increase in burden as patients progressed from GERD to dysplasia, with descriptively higher all-cause and disease-related HRU once they developed EAC, as would be expected. More than half of the patients in the EAC cohort (53%) had a disease-related hospitalization during an average study period of 17 months; 30% required an ED visit and 82% had an office visit. These findings are consistent with those of recent work by Abraham et al,^22^ which showed that 60% of newly diagnosed advanced EAC patients had at least 1 disease-related hospitalization during an average study period of 10 months, and 26% of patients had at least 1 disease-related ED visit.

The high healthcare service use contributed to high medical costs, driven particularly by the costs of IP admissions and OP visits, consistent with previous findings.^20,22^ In this study, total disease-related medical costs incurred per year increased numerically from $6765 in the GERD cohort to $23 589 in the HGD cohort in an incremental fashion; in relation, costs incurred by patient in the EAC cohort were substantially higher at $145 302 per year. While no other study has previously provided a stepwise evaluation of the incremental costs incurred by disease stage, our results align with the healthcare expenditures previously reported for some of the cohorts. For example, Sarvepalli et al^23^ found that IP costs for patients with EAC reached as high as $67 503 in 2010; in our study, disease-related IP costs amounted to a mean (SD) of $72 353 ($185 685) per year for patients in the EAC cohort. Notably, we observed an increasing importance of IP costs with disease progression, which is expected as more complex care, including hospitalizations, are common among patients with cancer and constitute the majority of costs incurred during end-of-life care.^24,25^ Consistent with the findings of Brook et al,^20^ OP costs in this study accounted for a large proportion of costs, with 60% to 70% of total medical costs for BE patients with dysplasia, and between 40% to 60% across other cohorts. The results of the current work underscore the significant burden in this patient population and can serve as a benchmark to assist in improving their clinical management and inform future economic evaluations.

While the direct medical costs incurred by patients with esophageal disorders can be significant, indirect costs also constitute an important proportion of total costs.^11^ Although this burden was not evaluated in the current study, several groups have previously reported on loss of work productivity and lower health-related quality of life associated with GERD and BE.^21,26–28^ Additionally, research suggests that patients with EAC are often diagnosed at an advanced stage where curative treatment is no longer effective.^29^ Indeed, despite current screening and surveillance guidelines, one study found that only 12% of EAC patients had a previous history of BE.^30^ Considering the dismal clinical outcomes among patients with EAC and the substantial incremental cost burden observed with disease progression from GERD to EAC, these findings highlight the importance of screening and surveillance in this population, which has the potential to prevent disease progression and reduce the associated disease burden.

Our study has several strengths as well as shortcomings. This large, retrospective cohort study provides a comprehensive up-to-date report of HRU and healthcare costs associated with GERD, BE, and BERN and the findings contribute novel information on the healthcare burden in the United States. However, as it was a claims-based analysis, it is subject to common inherent limitations. Although the identification of patients with GERD, BE, and BERN in the United States using claims data has been previously conducted in published literature,^16–18^ the claims-based algorithm used in this study has not been validated. The medical claims database included only diagnosis, procedure, or treatment codes that were recorded for reimbursement purposes. As laboratory test results were not available, the identification of cohorts was mainly based on diagnosis codes in medical claims, and treatments were identified based on pharmacy claims for a filled prescription. Since over-the-counter treatments are not available in claims, self-management of the disease was not captured. Furthermore, the reasons for diagnosis or procedure codes and information on socioeconomic factors and death, are not available in claims data. Claims data may be subject to omissions and inaccuracies. However, claims data used in this study allowed us to comprehensively assess HRU and costs incurred by patients ranging from GERD to BE to BERN. Finally, the study sample was limited to commercially insured patients with a slightly higher representation from the South and North Central (Midwest) regions. As such, the generalizability to the overall US population may be limited by this aspect of the study design.

## CONCLUSIONS

This large, retrospective, claims-based cohort study showed that patients with GERD, BE, and BERN had important disease-related HRU and direct healthcare costs, most notably for office visits and IP admissions. As patients progressed from GERD to EAC, they incurred substantially higher resource utilization and costs. These descriptive findings provide insights of potential value to a range of real-world decisions on the allocation of resources and management of patients with GERD, BE, and BERN in the United States and suggest the need for early identification of high-risk individuals prior to progression to EAC to potentially improve clinical and economic outcomes in this population.

### Author Contributions

All authors have made substantial contributions to the conception or design of the study, or the acquisition, analysis, or interpretation of data, drafting the manuscript and revising it critically for important intellectual content; have provided final approval of this version to be published; and agree to be accountable for all aspects of the work.

### Disclosures

P.S. reports grants from Cosmo Pharmaceuticals, Covidien, Docbot, ERBE USA, Inc., Fujifilm Holdings America Corporation, Ironwood Pharmaceuticals, Inc., Medtronic USA, Inc., and Olympus, and consulting fees from Bausch, Boston Scientific Corporation, CDx Diagnostics, Covidien, Exact Sciences, Fujifilm Medical Systems USA. Inc., Lucid Diagnostics, Lumendi, Medtronic, Phathom Pharmaceuticals, Olympus, Takeda, and Samsung Bioepis, and served as Chair for American Society for Gastrointestinal Endoscopy (ASGE) AI Task Force, treasurer/executive committee member for American Society for Gastrointestinal Endoscopy (ASGE), and Chair for World Endoscopy Organization (WEO), Esophageal Disease Ad-Hoc Committee. G.W.F. reports research funding from Lucid Diagnostics and Castle Biosciences, and consulting fees from Castle Biosciences, CDx Diagnostics, Exact Sciences, and Lucid Diagnostics. A.B.O., M.M.E., and P.L. are employees of Exact Sciences and own stock and/or stock options. M.B. was an employee of Exact Sciences at the time the study was conducted. D.L.V., A.G., and S.S. are employees of Analysis Group, Inc., a consulting company that has provided paid consulting services to Exact Sciences, which funded the development and conduct of this study.

### Previous Presentations

Part of the material in this manuscript was presented in a poster at ISPOR (International Society for Pharmacoeconomics and Outcomes Research), Washington, DC, May 15-18, 2022.

## Supplementary Material

Online Supplementary Material
