# Supplementary material for: Healthcare Resource Utilization and Costs Among Patients With Gastroesophageal Reflux Disease, Barrett’s Esophagus, and Barrett’s Esophagus-Related Neoplasia in the United States
Source: J Health Econ Outcomes Res. 2023 Mar 3;10(1):51–8. doi: 10.36469/001c.68191 (PMC9985944; doi:10.36469/001c.68191)
Supplement: Online Supplementary Material [file jheor_2023_10_1_68191_151261.pdf]

Journal of Health Economics and Outcomes Research

Online Supplementary Material

Healthcare Resource Utilization and Costs Among Patients With Gastroesophageal Reflux Disease, Barrett's Esophagus, and Barrett's Esophagus–Related Neoplasia in the United States. *JHEOR*. 2023;10(1):51-58. doi:10.36469/jheor.2023.68191

Table S1: Annual Mean Number of HRU Events by Cohort from GERD to EAC Disease Stage

Table S2: Annual Direct Healthcare Costs by Cohort from GERD to EAC Disease Stage (USD 2020)

This supplementary material has been provided by the authors to give readers additional information about their work.

This is an open-access article distributed under the terms of the Creative Commons Attribution 4.0 International License (CCBY-4.0). View this license's legal deed at http://creativecommons.org/licenses/by/4.0 and legal code at http://creativecommons.org/licenses/by/4.0/legalcode for more information.

Table S1. Annual Mean Number of HRU Events by Cohort from GERD to EAC Disease Stage

	GERD (n = 3 310 385)	NDBE (n = 172 481)	IND (n = 11 516)	LGD (n = 4332)	HGD (n = 1549)	EAC (n = 11 676)
All-cause OP services,[a] PPPY; mean ± SD	18.92 ± 19.44	20.52 ± 19.88	21.47 ± 20.59	21.96 ± 20.40	23.27 ± 21.62	56.40 ± 43.34
Days with office visits[b]	8.46 ± 10.28	9.48 ± 11.26	9.85 ± 11.00	10.27 ± 11.91	10.55 ± 11.70	13.26 ± 12.72
Days with lab tests	4.29 ± 5.07	5.00 ± 5.36	5.01 ± 5.39	5.40 ± 6.07	5.84 ± 6.15	15.42 ± 14.10
Days with imaging services	2.62 ± 4.25	2.71 ± 4.54	2.78 ± 4.43	2.78 ± 5.49	2.84 ± 5.33	18.31 ± 24.95
Days with mental health services	1.46 ± 5.90	1.29 ± 5.68	1.53 ± 6.46	1.14 ± 4.65	1.10 ± 4.80	0.89 ± 3.62
Days with drug administration	2.29 ± 6.00	2.52 ± 5.90	2.59 ± 5.53	2.95 ± 5.61	3.68 ± 6.26	12.16 ± 16.07
Days with SNF	0.36 ± 4.09	0.18 ± 2.75	0.28 ± 3.42	0.21 ± 2.48	0.32 ± 3.48	1.23 ± 7.24
Days with home care/hospice services	0.95 ± 6.27	0.91 ± 5.70	1.09 ± 7.21	0.99 ± 5.55	1.18 ± 9.20	10.08 ± 24.86
Disease-related OP visits,[a] PPPY; mean ± SD	2.72 ± 3.62	3.48 ± 3.63	3.98 ± 5.24	4.54 ± 4.43	5.60 ± 5.17	34.12 ± 39.18
Days with office visits[b]	1.45 ± 2.14	1.55 ± 2.15	1.92 ± 2.61	2.05 ± 2.63	2.16 ± 2.81	6.27 ± 8.66

Abbreviations: EAC, esophageal adenocarcinoma; ED, emergency department; GERD, gastroesophageal reflux disease; HGD, high-grade dysplasia; HRU, healthcare resource utilization; IND, indefinite for dysplasia; IP, inpatient; LGD, low-grade dysplasia; NDBE, nondysplastic Barrett's esophagus; OP, outpatient; PPPY, per patient per year; SNF, skilled nursing facilities.

[a]OP services included office visits, laboratory tests, imaging, mental health services, drug administration, SNF, or home care services not received in an IP or ED setting.

[b]Office visits excluded days with OP services such as laboratory tests, imaging, mental health services, drug administration, SNF, and home care services.

Table S2. Annual Direct Healthcare Costs by Cohort from GERD to EAC Disease Stage (USD 2020)

	GERD (n = 3 310 385)	NDBE (n = 172 481)	IND (n = 11 516)	LGD (n = 4332)	HGD (n = 1549)	EAC (n = 11 676)
**All-cause costs, PPPY; mean ± SD (median)**						
Total	22 307 ± 64 644 (6871)	25 608 ± 64 954 (9532)	26 141 ± 61 967 (9686)	29 723 ± 56 548 (12 397)	44 192 ± 177 398 (19 729)	183 604 ± 249 111 (103 375)
Medical	18 559 ± 61 253 (4780)	21 261 ± 61 431 (6953)	21 642 ± 58 507 (6835)	24 916 ± 52 223 (9398)	39 234 ± 175 950 (15 985)	176 995 ± 247 601 (95 799)
IP	7046 ± 44 709 (0)	6961 ± 43 541 (0)	7392 ± 43 885 (0)	7144 ± 33 558 (0)	14 058 ± 166 185 (0)	83 222 ± 195 958 (17 072)
ED	1636 ± 7485 (0)	1490 ± 7 155 (0)	1604 ± 7806 (0)	1408 ± 5025 (0)	1694 ± 6294 (0)	4938 ± 14 209 (271)
OP	9877 ± 31 955 (3248)	12 810 ± 33 853 (5458)	12 646 ± 28 992 (5343)	16 363 ± 33 369 (7499)	23 482 ± 43 025 (12 325)	88 835 ± 123 586 (44 870)
Pharmacy	3748 ± 14 622 (614)	4347 ± 15 536 (792)	4499 ± 14 393 (841)	4807 ± 14 664 (1064)	4958 ± 13 687 (1363)	6609 ± 18 705 (1981)
**Disease-related costs, PPPY; mean ± SD (median)**						
Total	6955 ± 35 880 (1032)	8755 ± 34 224 (2676)	9675 ± 38 500 (2498)	12 241 ± 32 264 (4180)	24 239 ± 166 417 (8749)	146 319 ± 230 329 (63 627)
Medical	6765 ± 35 836 (849)	8473 ± 34 165 (2403)	9371 ± 38 445 (2226)	11 844 ± 32 186 (3758)	23 589 ± 166 347 (8069)	145 302 ± 230 112 (62 693)
IP	3410 ± 32 779 (0)	3141 ± 30 959 (0)	3872 ± 34 831 (0)	3347 ± 25 245 (0)	9585 ± 164 666 (0)	72 353 ± 185 685 (6801)
OP	2903 ± 11 926 (539)	5011 ± 11 780 (1993)	5092 ± 13 030 (1754)	8185 ± 18 844 (3117)	13 448 ± 20 486 (6748)	70 160 ± 109 069 (25 387)
ED	452 ± 4146 (0)	321 ± 3099 (0)	407 ± 4162 (0)	311 ± 2138 (0)	555 ± 3480 (0)	2789 ± 11 719 (0)
Pharmacy	190 ± 1046 (1)	282 ± 1373 (4)	303 ± 1440 (6)	397 ± 1546 (26)	650 ± 3120 (52)	1016 ± 4073 (24)

Abbreviations: EAC, esophageal adenocarcinoma; ED, emergency department; GERD, gastroesophageal reflux disease; HGD, high-grade dysplasia; HRU, healthcare resource utilization; IND, indefinite for dysplasia; IP, inpatient; LGD, low-grade dysplasia; NDBE, nondysplastic Barrett's esophagus, OP, outpatient; PPPY, per patient per year; USD, United States dollar.
